# The Treatment of Incipient Madness

**Published:** 1918-10-05

**Authors:** 


### THE TREATMENT OF INCIPIENT MADNESS.

Dr. Easterbrook, in his Report of the working of the Royal Crichton Institute, advocates, as has been repeatedly advocated in The Hospital, the extension of the system of admitting persons as voluntary boarders into lunatic asylums. At present this beneficial provision is granted to private patients but denied to rate-supported patients. Dr. Easterbrook asserts that those who are treated as voluntary boarders show a higher recovery rate than those who are certified; but the figures are far from convincing. Among the voluntary boarders the recovery rate was 53·4 per cent., while among the certificated patients it was only 43.1 per cent.; but obviously the voluntary boarders would be cases of a milder type of madness, if indeed they could rightly be called mad at all; and the two sets of cases are not comparable. The wonder is that the discrepancy is not much wider.

There is a strong demand at the present time by alienists for institutions in which incipient madness and mild cases of madness can be treated without resort to certification. The demand rests upon the old and often exploded fallacy that everything can be set right by legislation. Before new institutions are created for the treatment of incipient madness, why not utilise the institutions we already have? If the cases are treated early enough, they can very well be treated as out-patients, and no legislation nor any new institution is necessary for the treatment as out-patients—or as in-patients either—of persons who are in danger of becoming insane, or are in the incipient stage of madness. No appliances that are not to be found in every hospital are necessary for the treatment of such persons, and a clinic for mental diseases in every general hospital would suffice for their treatment. What is lacking is neither legislation, nor institutions, nor appliances, but physicians. It is not too much to say that there are no physicians competent to treat incipient madness; and the reason is plain:—There are no physicians who have any special experience in the study and treatment of this condition. Asylum physicians have no such experience, for they never see such cases. They do not see cases of madness until the disease is so fully established as to permit of certification, or so nearly established that the patient seeks refuge in an asylum as a voluntary boarder. The general physician and the general practitioner see only an occasional case, and hasten to pass on the care of it to someone else. Hence, there is no one competent to treat such cases, and they are not studied at all. If a special out-patient department were established for mental diseases in every general hospital, and a young and able physician were placed in charge of it, he would soon acquire enough experience to be able to treat these diseases competently. At present there is no one competent to do so.

It is a great mistake to suppose that every case of incipient madness, or, for the matter of that, of fully developed madness, is a fit and proper case to be put to bed. Thirty years ago there was a great outcry for the hospitalisation of asylums, and for the "hospital treatment" of the insane. It was a vague aspiration, and when its advocates were cross-questioned as to their meaning and pinned down to a plain statement, it came to this: that every patient who entered an asylum must be forthwith put to bed. In some asylums this was done. In some asylums it is done now. In some asylums a costly receiving ward has been built for the purpose. But no rise in the recovery rate has followed. It did not occur to the advocates of the "hospital treatment of insanity" that even some bodily diseases can be treated without necessarily putting the patient to bed. We do not send to bed every case of skin disease, of disease of the eye, or ear, or throat, or even of more general disease, such as fibrosities, or rheumatoid arthritis or arterio-sclerosis. No. Decubitisation, as we may call it, is a useful measure, no doubt, in acute general disease, but it is not, any more than any other measure, a panacea for every disease to which mankind is subject, and putting the patient to bed, taking his temperature (which is almost always normal), and placing him on milk diet, which are what the advocates of "the hospital treatment of insanity" have in their minds, will not cure insanity any more than they will cure deafness. It is as well sometimes to clear our minds of cant.

